# The Administration of Drugs

**Published:** 1868-12

**Authors:** 


### The Administration of Drugs.

The following very excellent suggestions on the administration of medicines occur in an address of Dr. T. King Chambers, delivered at the recent meeting of the British Medical Association:

"I wish to offer a few suggestions concerning the administration of medicines, which may tend to make this daily business of ours more available in increasing the therapeutical science: (1.) Let us aim at giving only *one drug at a time.* I do not say this is always possible; but at all events let us keep the desire in our minds, and reckon a prescription good in an inverse ratio to the number of ingredients. This simplicity conduces not only to the good of science, but of our individual patients, for it soon makes us much more ready at suiting the special remedy to the special case. (2.) It is important, when we change our treatment, to allow a certain sufficient interval, different in different instances, between leaving off one medicine and beginning another. The experiments of Böcker and others have shown us, first, an action of the drug lasting after its apparent disappearance from the body, and, secondly, a reaction of the system opposite to, though weaker than, the original action. Advantage will accrue to the patient often from this rule, too. For instance: hyoscyamus, given for hypochondriasis or mental depression, may be left off almost directly it has begun to produce its beneficial effects, and those beneficial effeets will still go on towards restored health. Hydrochlorate of strychnia will continue to invigorate the peristaltic motions of the stomach and intestines, so as to produce steady digestion and evacuation, for days after such a soluble salt must have passed away. (3.) It is advisable for each observer to have as short a pharmacopœia as possible. The best workmen use the fewest tools—aye, and those who use the fewest tools become the best workmen. They become more adroit with them, know them better, and are able to instruct others in their employment. (4.) The union and coöperation together of those who are working at the same subject are of incalculable value. Incalculable—because you have not tried it. The skeleton of the machinery exists in the British Medical Association. Why should not each branch or group of branches take up a drug, and let us know after two or three years their experience of its action?"

This would result in some real and practical knowledge of the action and value of medicines, and not, as at present, depend on supposed and theoretical traditions.—*St. Louis Medical Reporter.*

